# Towards the Development of a Female Animal Model of T1DM Using Hyaluronic Acid Nanocoated Cell Transplantation: Refinements and Considerations for Future Protocols

**DOI:** 10.3390/pharmaceutics13111925

**Published:** 2021-11-13

**Authors:** Fernanda Zamboni, Ibrahim F. Cengiz, Ana M. Barbosa, Antonio G. Castro, Rui L. Reis, Joaquim M. Oliveira, Maurice N. Collins

**Affiliations:** 1 Stokes Laboratories, School of Engineering, Bernal Institute, University of Limerick, Limerick V94 T9PX, Ireland; fernanda.zamboni@ul.ie; 2 Health Research Institute, University of Limerick, Limerick V94 T9PX, Ireland; 3 13B’s Research Group, I3Bs—Research Institute on Biomaterials, Biodegradables and Biomimetics, University of Minho, Headquarters of the European Institute of Excellence on Tissue Engineering and Regenerative Medicine, AvePark, Parque de Ciência e Tecnologia, Zona Industrial da Gandra, 4805-017 Barco, Guimarães, Portugal; fatih.cengiz@i3bs.uminho.pt (I.F.C.); rgreis@i3bs.uminho.pt (R.L.R.); miguel.oliveira@i3bs.uminho.pt (J.M.O.); 4 ICVS/3B’s—PT Government Associate Laboratory, 4710-057 Braga, Braga, Portugal; id7167@alunos.uminho.pt (A.M.B.); acastro@med.uminho.pt (A.G.C.); 5 Life and Health Sciences Research Institute (ICVS), School of Medicine, University of Minho, Campus Gualtar, 4710-057 Braga, Braga, Portugal; 6 SFI AMBER, University of Limerick, Limerick V94 T9PX, Ireland

**Keywords:** diabetes induction, female animal model, transplantation

## Abstract

Female mice (Black 6 strain) (C57BL/6) aged 6 weeks were subject to low dose streptozotocin (STZ) treatment for five consecutive days to mimic type 1 diabetes mellitus (T1DM) with insulitis. At two weeks after STZ injections, evaluation of the elevated glucose levels was used to confirm diabetes. The diabetic mice were then subject to the transplantation of pancreatic β-cells (MIN-6 line). Four groups of mice were studied. The first group was injected with saline-only acting as the placebo surgery control, also known as SHAM group, the second and third groups were injected with MIN-6 single cells and polyethylene glycol-modified dipalmitoyl-glycerol-phosphatidyl ethanolamine (PEG-DPPE) modified MIN-6 single cells (500 µg per 1.10^6^ cells), respectively, while the fourth group was injected with hyaluronic acid (HA)-coated MIN-6 single cells (5 bilayers). At seven- and fourteen-days following transplantation, the mice were euthanised. The renal and pancreatic tissues were then collected and histologically analysed. The induction of diabetes in female mice, through five-consecutive daily STZ injections resulted in inconsistent glycaemic levels. Interestingly, this shows an incomplete diabetes induction in female mice, of which we attribute to sex dimorphism and hormonal interferences. Transplantation failure of free-floating encapsulated cells was unable to decrease blood glucose hyperglycaemia to physiological ranges. The result is attributed to deprived cell–cell interactions, leading to decreased β-cells functionality. Overall, we highlight the necessity of refining T1DM disease models in female subjects when using multiple low-dose STZ injections together with transplantation protocols. Considerations need to be made regarding the different developmental stages of female mice and oestrogen load interfering with pancreatic β-cells susceptibility to STZ. The use of pseudo islets, cell aggregates and spheroids are sought to improve transplantation outcome in comparison to free-floating single cells.

## 1. Introduction

Many animal studies use pancreatomy to induce a state of absolute insulin deficiency and hyperglycaemia in order to mimic diabetes [1]. However, many other hormones (e.g., glucagon) and digestive enzymes are also extinguished when performing a total pancreatomy. Diabetes-inducing agents such as STZ selectively obliterates insulin-producing cells in the pancreas while maintaining the remaining functionality of the organ. STZ is a broad-spectrum antibiotic with unique toxic selectivity for β cells in the pancreas, being used clinically for the treatment of metastatic insulinomas. STZ uptake into rodent pancreatic β cells is mediated by GLUT2 receptor. STZ is equally diabetogenic whether administered to fed or fastened mice [2], showing no competition with glucose for the GLUT2 receptor.

Outside its therapeutic application, STZ has been used since early 1960s in diabetes research in order to induce hyperglycaemia in rodents via selective destruction of β cells. This effect is attributed to the nitroso moiety in STZ. Once inside the cell, this nitroso group acts as a nitric oxide (NO) donor to create reactive oxygen species and induce cell death [3].

However, the cell death pathway greatly differs by the STZ posology adopted for the induction of hyperglycaemia in mice. When delivered by intraperitoneal injection as a single high dose (180–190 mg/kg body weight), STZ causes massive β-cell necrosis within 2–3 days after administration. One moderate dose (150 mg/kg body weight) in conjunction of high fat diet can also accelerate β-cell endoplasmic reticulum stress which mimics type 2 diabetes mellitus with high toxicity and mortality rate. Whereas multiple low doses of STZ mimics T1DM with insulitis [2].

STZ protocols for diabetes induction produce a reliable T1DM animal model, specifically applied in pre-clinical research related to the transplantation of allogenic and xenogeneic pancreatic β cells. However, from the array of immortal pancreatic β-cell lines available for transplantation, MIN-6 cells originated from transgenic C57BL/6 mouse insulinoma and capable of expressing an insulin-promoter/SV40 T-antigen construct are one of the few immortal cell lines to retain glucose-stimulated insulin release [4]. The transplantation of these cellular grafts embedded in a cell encapsulation coating is key to protect pancreatic β cells from the host immune system [5], while maintaining cell functionality and physiological insulin secretion patterns in response to blood glucose concentrations [6].

However, animal research across all disciplines predominantly uses male subjects instead of mixed or only female subjects. The incorporation of only male subjects in animal research is likely to lead to poorer treatment outcomes for women in the future, as studies have already revealed marked differences between male and females in many basic biological processes [7]. The National Institute of Health (NIH) in the USA has been developing policies encouraging the use of both male and female animal research subjects and consideration of sex and biological variables [8].

In the context of T1DM pre-clinical research, when male and female mice are transplanted with two different stages of pancreatic cells derived from human embryonic stem cells (endocrine progenitors and insulin-positive cells), the in vivo maturation of both cell populations into insulin-secreting cells was accelerated in female recipients. The oestradiol-2 (E2) hormone was the promoter of a faster β cells maturation [9].

In humans, women tend to have an increased glucose-stimulated insulin secretion (GSIS) due to a higher GLP-1 production associated to the female E2 hormone [9]. E2 hormone also influences the immune system, which plays a central role in the development of T1DM. The immunological effects of E2 on innate immune system show to be protective against innate immune pro-inflammatory responses and prevents apoptosis of islets. E2 also protect islets from the adaptive immune response, where E2 hormone promotes the expansion of immunosuppressive Treg cells. In ketosis-prone diabetes (KPD), which is a form of type 2 diabetes mellitus, a male predominance is also observed. Interestingly, the rare women developing KPD were in an anovulatory state, with decreased E2 levels [9].

Endocrine pancreas cell composition is known to vary according to pancreatic anatomical location and amongst different species [10]. Moreover, the proportion of different hormone-producing cells in the pancreas also differs between males and females of the same species, possibly also contributing to the female resistance to diabetes induction. Studies suggest that islets from women have an average of 6% more β cells than men, moreover, islet transplantation from female donors also show better outcomes than with islets obtained from male donors [9].

In this context, sex dimorphism and hormonal variances may interfere with the inducing effects of STZ. Herein, we assess the suitability of a T1DM protocol utilizing low-dose STZ injections using solely C57BL/6 female mice subjects in a post-puberty developmental stage, where mice present oestradiol hormones produced during the oestrous cycle. The transplantation of encapsulated free-floating MIN-6 cells was also performed with the ultimate aim of providing a pathway to decrease hyperglycaemia and reverse diabetes in this female animal model that should resemble autoimmune T1DM.

## 2. Materials and Methods

### 2.1. Materials

HA, with an average molecular weight (MW) of 1.20 MDa and 0.1 MDa, was kindly supplied by Shanghai Easier Industrial Development Co. LTD. (Shanghai, China) as dry powder. Maleimido propionyl-polyethylene glycol-n-hydroxysuccinimide ester (Mal-PEG-NHS), dipalmitoyl-glycerol-phosphatidyl ethanolamine (DPPE), 2,2′-dithiodipyridine, dithiothreitol (DTT) and phosphate buffered saline (PBS) were purchased from Sigma Aldrich (St. Louis, MO, USA). All other consumables necessary for cell culture including cell culture medium, pipettes, flasks and plates were purchased from Thermofisher. Pancreatic MIN-6 cells were provided by the I3Bs Research Group at the University of Minho, Portugal.

### 2.2. Animals

Female mice of Black 6 strain (C57BL/6) aged 6 weeks were housed in ventilated cages of no more than 5 mice per cage with free access to food and water, at a controlled temperature of 23 °C with 12 h light-dark cycles. All procedures were performed in accordance with the European Directive 86/609/EEC and approved by the University of Minho Animal Ethics Committee (SECVS 074/2016) and the Portuguese National Authority (DGAV 014072).

### 2.3. Model for Diabetes Induction

All mice were subjected to low dose STZ treatment to mimic T1DM with insulitis. An STZ solution was freshly prepared using sodium citrate pH 4.5 prior to be administered intraperitoneally (50 mg/kg of body weight) once a day for five consecutive days. At the time of the injections, mice were kept ad libitum fed. Diabetes development was assessed by measurement of blood glucose levels (Freestyle Precision Neo, Abbott).

### 2.4. Conformal Cell Coating

**Figure 1:**
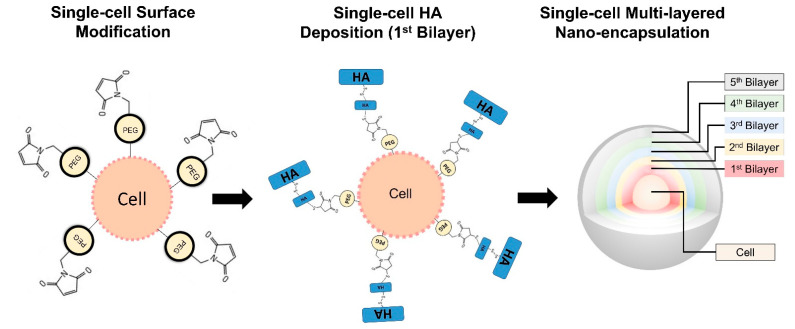
Schematic representation of free-floating single cell encapsulation. Surface modification using Mal-PEG-DPPE and deposition of multiple layers of HA derivates.

MIN-6 cells, 30–40 passages, were cultured in a density of 10 × 10^6^ cells/flask in high glucose DMEM, supplemented with 10% FBS, 1% antibiotic/antimycotic, 10 mM sodium pyruvate and 50 µM β-mercaptoethanol. Cell media was replenished every 3 days until cell confluency was reached. Cells were trypsinised and the resulting free-floating single cells were further used for different encapsulation processes (Figure 1).

Free-floating single MIN-6 cells at a density of 1 × 10^6^ were incubated with Mal-PEG-DPPE at 500 µg.mL^−1^ for 30 min. Mal-PEG-DPPE synthesis is described elsewhere [11]. After, cells were centrifuged and washed with PBS 1X, resulting in PEG-DPPE modified MIN-6 single cells. After washing, cells were centrifuged and the supernatant was removed. Cells were incubated in solutions containing 1.8 mg × mL^−1^ of different HA derivates in DMEM. Synthesis of HA derivates carrying free thiol groups (HA-SH) and pyridine groups (HA-PD) has been described elsewhere [12,13]. The first conformal layer was deposited onto the surface of the cells by incubating HA-SH 0.1 MDa for 10 min. After the cells were centrifuged and washed with PBS 1X, they were incubated with HA-PD 1.2 MDa for 10 min. This first cycle is the first coating bilayer. For the second bilayer, cells were incubated with HA-SH 0.1 MDa for 10 min. Then the cells were centrifuged and washed with PBS 1X and were incubated with HA-PD 1.2 MDa for 10 min. For the third bilayer, cells were incubated with HA-SH 1.2 MDa for 10 min. Then the cells were centrifuged and washed with PBS 1X and incubated with HA-PD 1.2 MDa for 10 min. For the fourth and fifth bilayers, cells were incubated with HA-SH 1.2 MDa for 10 min. Then the cells were centrifuged and washed with PBS 1X and incubated with HA-PD 1.2 MDa for 10 min.

### 2.5. MIN-6 Graft Transplantation

Diabetic mice were subject to the transplantation of pancreatic β cells (MIN-6 line). Four groups of mice were used. Mice injected with saline-only (SHAM group), mice injected with MIN-6 single cells, mice injected with PEG-DPPE modified MIN-6 single cells and mice injected with HA-coated MIN-6 single cells.

Prior to the surgery, intramuscular (IM) injection (100 µL) of 90 mg/kg ketamine (Imalgen; Meriel, Lyon, France) in combination with 0.65 mg/kg of the α2 adrenergic receptor agonist medetomidine (Domitor; Pfizer, Paris, France) were used for anaesthesia and sedation, respectively. Shaving and incision under the left rib was performed. A cell suspension of 750,000 cells per mice or 10 µL of saline was injected under the left kidney. Mice were sutured and they were given an IM injection (50 µL) of atipanezole 1 mg/kg (Antisedant; Pfizer, Paris, France) as a sedative reverser. Mice were put to rest under warm light.

### 2.6. Blood Glucose Control

Mice were assessed for up to 14 days post-transplantation to analyse diabetes reversion through the measurement of blood glucose levels twice a week. Blood samples were collected from pricking the mice cheek with a needle. The blood droplet was placed in a glucose strip and measured using a glucometer (Freestyle Precision Neo, Abbott). After follow-up, mice were sacrificed for histological analysis

### 2.7. Dissection of Renal and Pancreatic Tissues

Mice were euthanised via CO_2_ inhalation at different time points. 7 days and 14 days of after transplantation mice from each group (n = 5) were sacrificed for the collection of renal and pancreas tissues to determine the leukocytic infiltration degree. Renal and pancreatic tissues were retrieved and fixed using 4% formalin solution. Fixed kidneys and pancreas were embedded in paraffin and cut in 5 µm thick sections using a microtome.

### 2.8. Histology

Haematoxylin and eosin were used to analyse graft-localised cell infiltration and inflammation. All images were acquired using a CKX41 Olympus inverted microscope (Tokyo, Japan) equipped with a DFK 31AU03 camera (The Imaging Source Europe GmbH, Germany) and IC Capture software (The Imaging Source Europe GmbH, Germany).

### 2.9. Statistical Analysis

Data are presented as mean ± standard deviation (s.d.) and analysed using one-way analysis of variance (ANOVA) followed by post-hoc Tukey’s HSD test. *p*-values < 0.05 (*) were considered significant.

## 3. Results and Discussion

### 3.1. Animal Model for Diabetes Induction

Diabetes was induced in female mice via 5 consecutive low-dose STZ injections (Supplementary Figure S1). The blood glucose levels of the female mice after 14 days of the injections are shown in Table 1. The blood glucose levels of 20% of all mice subjected to the induction were unable to achieve hyperglycaemia after 14 days of the last STZ injection, with blood glucose levels falling between 100 and 149 mg/dL. By its turn, 32% of the mice displayed little increase in blood glucose levels (150–199 mg/dL), however still falling into a non-diabetic category. Moreover, 48% of the mice were successfully induced with diabetes, showing high blood glucose levels above 200 mg/dL (hyperglycaemia). These findings highlight that diabetes induction in female mice is associated with sexual dimorphism assigned to the oestradiol hormone.

Sex differences in physiology begin early in development, due to a combination of genetic and hormonal cues which continue after puberty. In multiple rodent models of diabetes in which β cell failure occurs, sex dimorphism is also observed. It is known that female mice will develop insulitis, with modest hyperglycaemia (around 200 mg/dL) 14 days following the STZ injections. While male mice develop diabetes with severe hyperglycaemia (>400 mg/dL) during the course of the 5 days of injection [14,15]. These differences are assigned to oestradiol, the major female oestrogen steroid hormone, which is presumed to protect the β cell from oxidative stress-induced apoptosis [16]. The protective effect of oestradiol in the β cells against oxidative stress is believed to be mediated by three oestrogen receptors expressed in β cells, ER-alpha, ER-beta and G-protein coupled oestrogen receptor [17].

**Figure 2:**
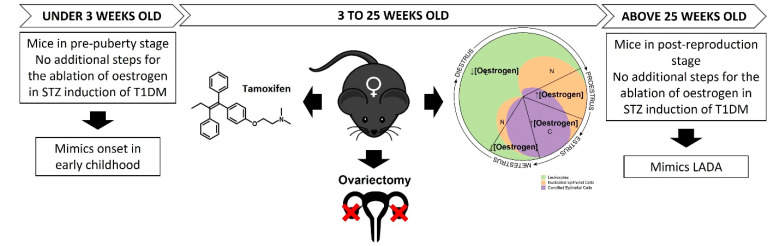
Proposed approaches to refine T1DM disease models using multiple low-dose STZ injections. Considerations regarding the different developmental stages of female mice and oestrogen load are highlighted.

In this study female mice aged 6 weeks old were used to recreate a T1DM model. At this age mice already achieved puberty and are considered adults, thus the oestrogen levels in the female mice play a role in the decreased efficiency of STZ induction [18]. Although the results shown in this project have great variability regarding diabetes induction and hyperglycaemia achievement, it corroborates that more research and adaptation of current protocols have to be made in order to better characterise disease models in female subjects (shown in Figure 2).

We speculate that, for mice aged <3 weeks which are in a pre-puberty, the induction of diabetes can potentially be more reliable as these mice are not in their reproductive cycle yet, thus the oestrogen dependant effect on STZ would not be present. On this point, it is worth noting that in humans T1DM onset starts in early childhood [19], and utilizing mice at a pre-puberty stage is a good strategy to reproduce onset in early childhood.

However, T1DM induction in female mice over 3 weeks old is possible with the ablation of the oestrogen hormone. In this scenario, ovariectomy could be implemented together with STZ induction [20]. However, this method of oestrogen ablation is known to produce important side effects. So as an alternative, STZ induction could be potentially administered concomitantly with oestrogen receptor antagonists, like tamoxifen, for the ablation of oestrogen in female mice at reproductive stages [21].

Moreover, the great variability of hyperglycaemia within female mice (reported in Table 1) is sought to be a consequence of each individual rodent reproductive cycle. In rodents the reproductive cycle, called the oestrous cycle, lasts approximately 4–5 days (in humans it is called the menstrual cycle, and lasts approximately 28 days). The oestrous cycle has four stages (proestrus, oestrus, metestrus and dioestrus). Each stage lasts for approximately one day [22]. Proestrus stage corresponds to the pre-ovulatory day, when E2 increases and consequently, during the night, luteinizing hormone (LH) and follicle-stimulating hormone (FSH) surge and ovulation occurs. In the oestrus stage, E2 remains elevated throughout the morning and falls back to basal levels in the afternoon. In the metestrus stage, plasma E2 concentration is low. Finally, in the dioestrus stage, E2 levels start to increase [23]. Considering that 52% of all mice did not achieve hyperglycaemia, and 48% of the mice were successfully induced with diabetes, showing high blood glucose levels, it may be possible that the start of the oestrous cycle (high E2 levels) coinciding with the start date of STZ injection, decreases the efficacy of STZ induction. While starting the STZ induction in the metestrus stage, where low levels of E2 are found, may increase STZ-induced cell toxicity and higher diabetes induction efficiency rates.

In humans, a further subtype of T1DM affects an older demographic group known as Late Onset Autoimmune Diabetes of Adults (LADA) [24]. In recent epidemiological studies, T1DM in children has shown no gender predilection, due to the pre-puberty lack of hormone interference at disease onset. However, in LADA, where the average onset T1DM is at the age of 40 years old, there is a significant difference (*p* < 0.001) in gender-prevalence. At this age group, LADA has been shown to affect 80% of males and only 20% of females. Showing that women are less likely to develop LADA in their reproductive years (levels of oestrogen around 400 pg/mL) up to their pre-menopausal stage. However, it is noted that after the age of 45 years old, onset of LADA decreases its incidence in men to 40%, but skyrockets in women (60%) [25]. Once women reach menopause age and the oestrogen levels drop below 30 pg/mL, pancreatic β-cell populations are more susceptible to cell death. In this regard, the use of post-reproductive female mice subjects (aged above 750 days) may be considered to increase hyperglycaemic state after multiple low-dose STZ injections in T1DM models [18].

### 3.2. In Vivo Transplantation in the Renal Subcapsular Space

Deviation in mice, the kidneys are located retroperitoneally, where the left kidney is readily visible and can be accessed more easily in comparison to the right kidney, which is located under the small intestine and adjacent to the liver [26]. In the kidneys, the adrenal glands are in close proximity to each renal cranial pole, and they can be distinguished by the light orange coloration due to steroid hormone production [27]. The kidneys in rodents are unilobular, unipapillary and unipyramidal, in contrast to human kidneys which are multilobular, multipapillary and multipyramidal. On a cross-section of the kidney, four main structures can be seen: the capsule, the cortex, the medulla and the papilla which extend deep into the renal pelvis. The capsule is made of epithelial and fat tissue. In the renal cortex, the glomeruli (with an average diameter of 73.4 µm) and the proximal convoluted tubules can be found. In the medulla (pyramid) the medullary capillary plexus is found. Little interstitial connective tissue is found in the kidney of rodents in contrast to kidneys of humans [26]. In this study MIN-6 cells were injected under the sub-capsule membrane of the kidney as single-cell treatments as shown in Supplementary Figure S2.

**Figure 3:**
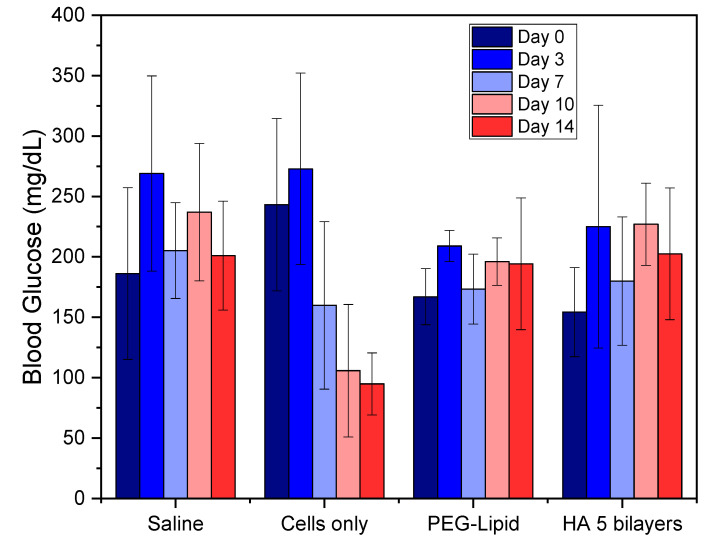
Post-transplantation blood glucose levels for mice groups euthanised at day 14. Sample size n = 5 for each group.

Five mice have died at day 3 post-transplantation. Of the ten mice for each group, three mice died from the group implanted with PEG-DPPE cell surface modification, one mouse died from the group implanted with HA encapsulated cells, and one mouse died from the SHAM group. These deaths are attributed to stitch opening and infection at the incision site. After the transplantation, mice had their blood glucose concentrations recorded for 14 days post transplantation (Figure 3).

**Figure 4:**
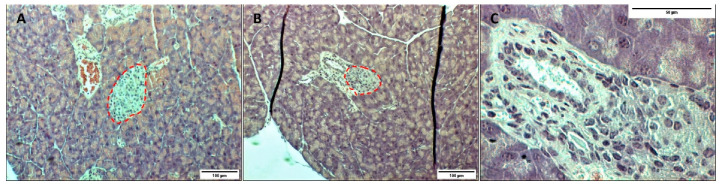
Histological images of pancreatic tissue from STZ-induced diabetic female mice on day 7 (**A**), and day 14 (**B**) after transplantation, where (**C**) depicts a higher magnification for day 14 after transplantation. Islets are circled in red. Tissues were stained using haematoxylin and eosin.

In Figure 3, although there is no significant difference in blood glucose levels for the different groups over time (apart from cells only group), it can be seen that at day 0 (prior to transplantation) all mice had reached mild hyperglycaemia (200–300 mg/dL), except for the cell surface modification group. However, if we observe the SHAM group on day 3 after transplantation, we can notice an ongoing rise in blood glucose levels. This result suggests that diabetes induced by STZ was not complete after 14 days as suggested by the literature [14,15]. On day 7, we can observe a slightly decrease of blood glucose level, which is associated with β-cell recovery due to an incomplete β-cell destruction [16]. When analysing the pancreas of mice, islets are unevenly distributed throughout the pancreas, and are present in both interlobular (Figure 4A) and periductal/perivascular (Figure 4B) locations [28]. In the islets of mice, β-cell populations are typically clustered in the centre of the islet and surrounded by other islet cell types, whereas in humans all islet cells are homogenously intermingled [29].

Regarding the morphology of the islets after treatment with STZ, their architecture is expected to be largely disrupted [30]. However, as seen in Figure 4C, islets did not show morphological changes nor boundary irregularities. Moreover, no infiltration of immune cells was observed in the histological analysis of the pancreas of STZ-induced diabetic mice. In this scenario, considering the results presented, diabetes induction cannot be assured only 14 days after STZ injections. It is recommended to consider extending the induction for at least 21 days after STZ injections or increasing the daily dose of STZ.

**Figure 5:**
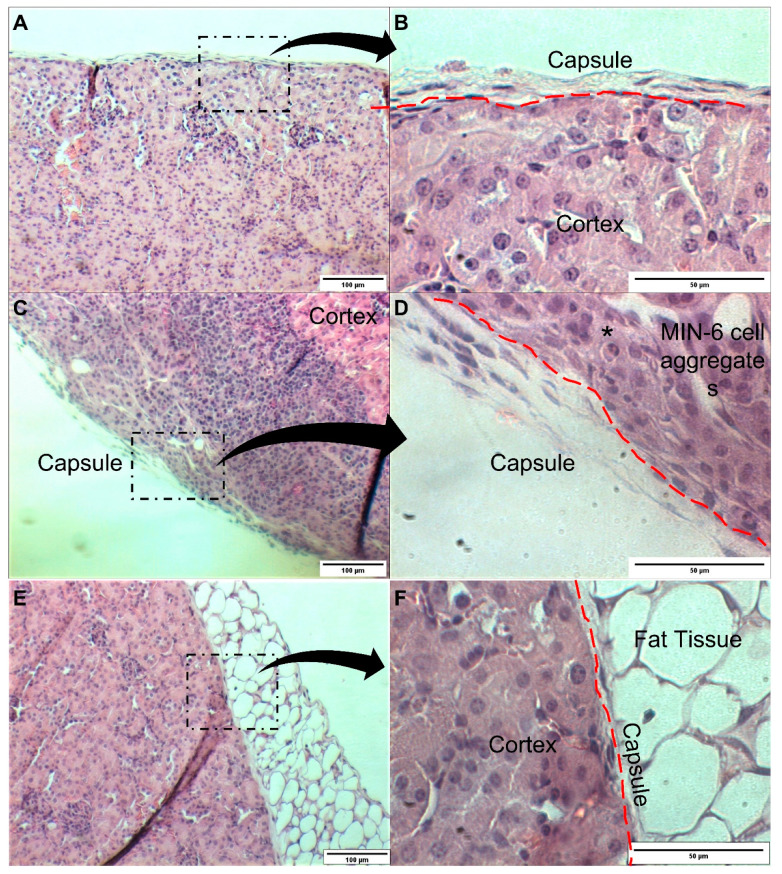
Histology analysis of the kidneys after 14 days post-transplantation of MIN-6 single cells. Saline group (**A**,**B**), cells only group (**C**,**D**), and encapsulated cells (**E**,**F**). Immune cell infiltration is shown (*). Lower magnification 10×, scale bar 100 µm. Higher magnification 40×, scale bar 50 µm.

Furthermore, in Figure 3, results show that non-encapsulated single cells (cells only group) were able to decrease hyperglycaemia over time to normal physiological levels (<200 mg/dL). This was not observed in mice transplanted with encapsulated and surface-modified cells. We hypothesise that non-encapsulated cells were able to interact with each other and were probably able to form clusters to mimic a pseudo-islet. This hypothesis has been confirmed in the histological analysis of the renal tissue after transplantation. In Figure 5A,B, the saline transplanted group (SHAM) shows the normal appearance of the renal capsule and the underlying renal cortex. In Figure 5C,D, the histological analysis of the mice transplanted with naked cells, shows that the MIN-6 cells are grouped and located between the renal capsule and the renal cortex. Images also show graft infiltration with leukocytes. Infiltration of immune cells have also been reported by other studies at the 2-week transplantation mark. The degree of leukocyte infiltration is maximum at the first two weeks after transplantation and subsequently decreases [28].

Unfortunately, MIN-6 cells were not able to be found in the histological images of various slices of the kidneys of mice transplanted with encapsulated and surface engineering cells (Figure 5E,F). This finding confirms our hypothesis that surface-modified and encapsulated cells were deprived from cell–cell interaction, unable to form clusters similar to islets. This has also implications in β-cell functionality. Functional loss (measured by insulin expression, insulin content and glucose-stimulated secretion) is correlated with a decreased cell–cell interaction [31]. Other studies have also demonstrated this pattern of functionality associated to MIN-6 cell aggregates and single cells [32]. Another important point to highlight is that MIN-6 cells are anchorage-dependent cells. When single cells are encapsulated, they are denied attachment to one-another or to surrounding ECM, which likely triggers anoikis.

## 4. Conclusions

It is known that diabetes is achieved when levels of blood glucose are higher than 200 mg/dL. Reversion of diabetes and transplantation success is defined as the ability to reach non-fasting blood glucose levels under 250 mg/dL within five days of transplantation. Graft rejection is defined as two consecutive measurements above 300 mg/dL in mice after normoglycemia is achieved. T1DM induction disease protocols have been established using male mice and results here show that these protocols do not demonstrate a viable option to induce diabetes in female mice. This result is hypothesised to be a consequence of sexual dimorphism characteristics and hormonal interferences. As a result of these findings, we suggest the following T1DM-inducing protocol modifications:

- Increase the daily dose of STZ to above 50 mg/kg.
- If maintaining the 50 mg/kg dose a day, it may be advisable to increase the number of induction days above the 5 consecutive daily injections;
- In order to guarantee complete diabetes induction without β recovery, the post-induction period should be increased from 14 days to 21 days;
- Assess the interference of the oestrous cycle and the levels of oestrogen (E2) in the efficacy of STZ induction of diabetes in female mice aged >3 weeks (post-puberty stage);
- Assess the feasibility of using female mice aged <3 weeks (pre-puberty stage) to induce diabetes using STZ, as levels of oestrogen are neglectable. At this stage, T1DM may be better represented, as in humans, T1DM onset generally occurs in early childhood.

Lack of cell–cell interaction influences transplantation outcome and diabetes reversion in this work, with deficient insulin secretion is unable to control hyperglycaemia. To confirm this hypothesis, we suggest the following:

- Development of pseudo-islet aggregates using MIN-6 cells;
- Encapsulation of pseudo-islet;
- Assessment of glucose-induced insulin secretion;
- Transplantation of encapsulated pseudo-islet;
- Histological assessment of pseudo-islet aggregates under the kidney capsule

## Figures and Tables

**Table 1:** Blood glucose levels of mice at 14 days after STZ induction and before transplantation.

Blood Glucose Level	SHAM	Cells Only	Cell Encapsulation	Cell with Surface Modification	Total (%)
100–149 mg/dL	2	2	2	2	20
150–199 mg/dL	4	1	3	5	32
200–299 mg/dL	2	4	3	1	25
300–399 mg/dL	1	3	2	1	18
≥400 mg/dL	1	0	0	1	5

## Data Availability

University of Minho Animal Ethics Committee (SECVS 074/2016) and the Portuguese National Authority (DGAV 014072).

## Supplementary Materials

The following are available online at https://www.mdpi.com/article/10.3390/pharmaceutics13111925/s1. Figure S1 (A) Intraperitoneal injection of STZ. (B) Mice are kept in cages with ad libitum fed regimen. Figure S2 MIN-6 cell transplantation. (A) Abdominal viscera of female mice. (B) Intramuscular administration of anaesthetics. (C) Topical application of Vaseline to protect eyes from drying during surgery, lateral hair removal for incision. (D) Graft injection inside the kidney capsule. (E–F) Suture and stiches. (G) Post-operation recovery.
