# Supplementary material for: Towards the Development of a Female Animal Model of T1DM Using Hyaluronic Acid Nanocoated Cell Transplantation: Refinements and Considerations for Future Protocols
Source: Pharmaceutics. 2021 Nov 13;13(11):1925. doi: 10.3390/pharmaceutics13111925 (PMC8621706; doi:10.3390/pharmaceutics13111925)
Supplement: Supplementary file 1 [file pharmaceutics-13-01925-s001.zip › pharmaceutics-1413625-supplementary.pdf]

pharmaceutics

MDPI

Supplementary Materials: Towards the Development of a Female Animal Model of T1DM Using Hyaluronic Acid Nanocoated Cell Transplantation: Refinements and Considerations for Future Protocols

Fernanda Zamboni, Ibrahim F. Cengiz , Ana M. Barbosa, Antonio G. Castro, Rui L. Reis, Joaquim M. Oliveira and Maurice N Collins*

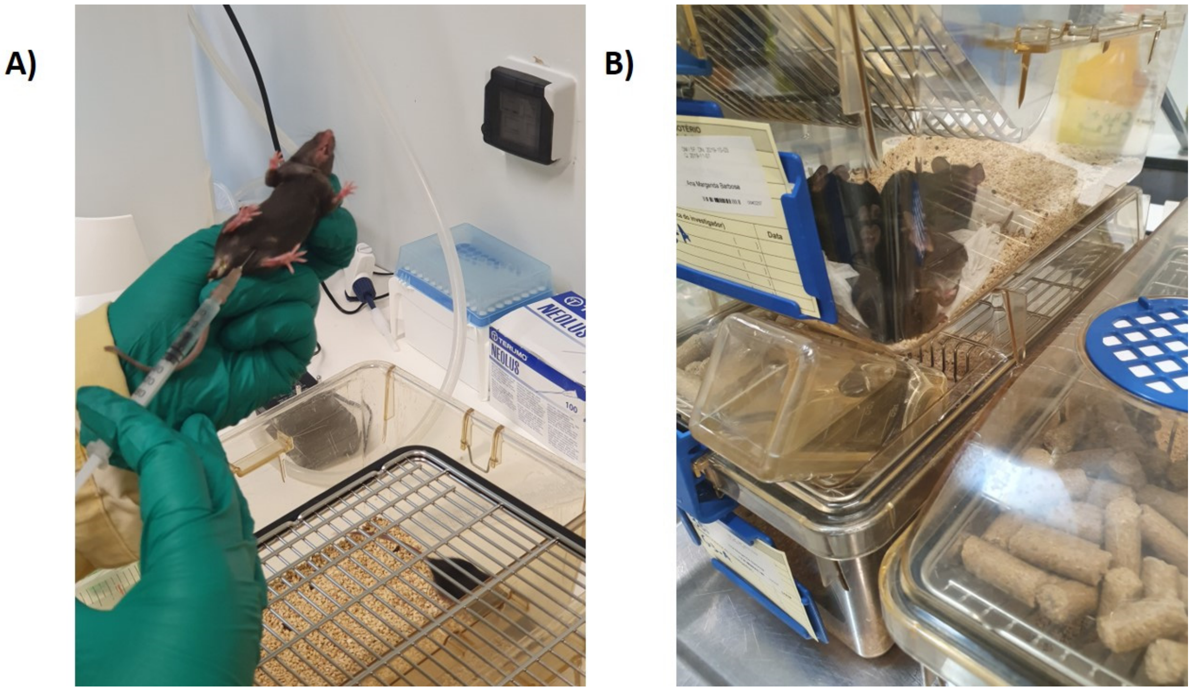

Figure S1. A) Intraperitoneal injection of STZ. B) Mice are kept in cages with ad libitum fed regimen.

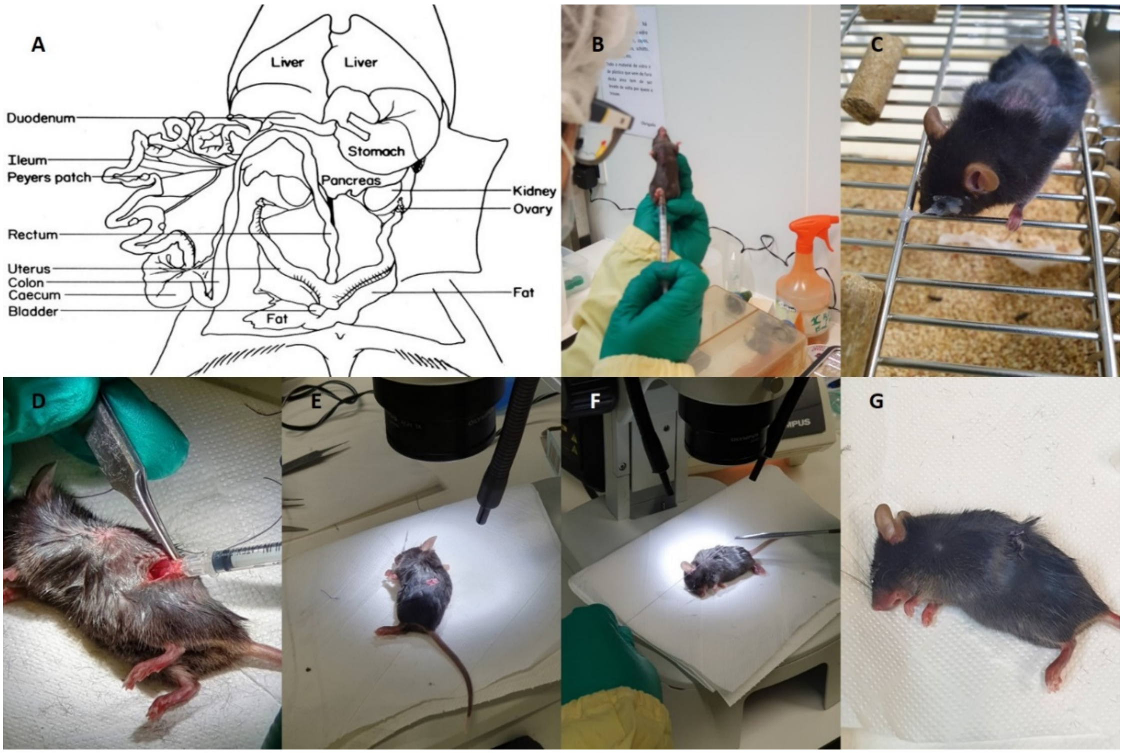

Figure S2. MIN-6 cell transplantation. A) Abdominal viscera of female mice [29]. B) Intramuscular administration of anaesthetics. C) Topical application of Vaseline to protect eyes from drying during surgery, lateral hair removal for incision. D) Graft injection inside the kidney capsule. E-F) Suture and stiches. G) Post-operation recovery.
